# PLANNED CESSATION OF POTENTIALLY INAPPROPRIATE MEDICATIONS: TRAINEE EXPERIENCES FROM LONG-TERM CARE PROGRAMS

**DOI:** 10.1093/geroni/igad104.0904

**Published:** 2023-12-21

**Authors:** Kimberly Dickerson, Jennifer Quellhorst

**Affiliations:** Central Arkansas Veterans Healthcare System, Little Rock, Arkansas, United States; WPB VAMC, West Palm Beach, Florida, United States

## Abstract

Polypharmacy is a global concern. There is no universal consensus on definition. VIONE is an innovative, end user friendly, portable, simple methodology that is easy to teach, adapt, customize across the continuum of care, in any clinical setting, for any medication. Trainees demonstrate immense eagerness, ability and potential to benefit from exposure to medication optimization through both safe prescribing and safe deprescribing practices. Given the good fortune of long term care settings, trainees follow the patients and directly engage in implementing methodical safe deintensification of potentially inappropriate medications through shared decision making. Trainees address prescription cascades and health literacy issues. Trainees also track outcomes to deprescribing, are made aware of the data and intervene as needed. Overall - this has been a very professionally rewarding and personally enriching experience. We have case reports with specific timelines, medications, doses, duration, rationale, responses and data analytics that illustrate the merits of engaging trainees early on in their careers to safe medication optimization and deprescribing practices. Data tracked includes number of patients impacted, number of medications deprescribed, dose decreased, safer alternatives prescribed, number of users, cost savings, location, medications deprescribed etc. that are updated daily with VIONE score cards and dashboards utilizing medical informatics and corporate data warehouse data - thus proving the rich granularity, transparency and data integrity.

